# Bidirectional Locomotion of Soft Inchworm Crawler Using Dynamic Gaits

**DOI:** 10.3389/frobt.2022.899850

**Published:** 2022-06-16

**Authors:** Liang Du, Shugen Ma, Keisuke Tokuda, Yang Tian, Longchuan Li

**Affiliations:** ^1^ Shanghai Robotics Institute, Shanghai University, Shanghai, China; ^2^ Faculty of Science and Engineering, Ritsumeikan University, Shiga, Japan

**Keywords:** soft robot, inchworm locomotion, bidirectional locomotion, friction force control, DEA

## Abstract

Inchworm-styled locomotion is one of the simplest gaits for mobile robots, which enables easy actuation, effective movement, and strong adaptation in nature. However, an agile inchworm-like robot that realizes versatile locomotion usually requires effective friction force manipulation with a complicated actuation structure and control algorithm. In this study, we embody a friction force controller based on the deformation of the robot body, to realize bidirectional locomotion. Two kinds of differential friction forces are integrated into a beam-like soft robot body, and along with the cyclical actuation of the robot body, two locomotion gaits with opposite locomotion directions can be generated and controlled by the deformation process of the robot body, that is, the dynamic gaits. Based on these dynamic gaits, two kinds of locomotion control schemes, the amplitude-based control and the frequency-based control, are proposed, analyzed, and validated with both theoretical simulations and prototype experiments. The soft inchworm crawler achieves the versatile locomotion result *via* a simple system configuration and minimalist actuation input. This work is an example of using soft structure vibrations for challenging robotic tasks.

## 1 Introduction

Soft robots are desirable novel robotic systems for their biomimetic properties and their deformable, lightweight, and energy efficiency superiorities over the traditional robots (Rus and Tolley, 2015; Gu et al., 2017). A lot of soft mobile robots have been proposed for various tasks, such as inspection, exploration, or even material transportation. Specifically, the inspection task in confined spaces requires the robot to adapt to strict environment constraints and pass through challenging obstacles, for example, in a disaster site (Hawkes et al., 2017), in an underwater field (Marchese et al., 2014), or even inside the human body (Adachi et al., 2011). For these purposes, a simple robot structure with strong locomotion capability will be crucial to deal with more challenging task requirements, that is, a simple system structure will ensure endurance and robustness, and a strong locomotion capability will enable access (getting in and getting out) to narrow spaces (Joey et al., 2019).

Inchworm locomotion, by cyclically contracting and extending two sides of the robot body, is one of the simplest locomotion forms for soft robots. A lot of research studies have been conducted to generate inchworm locomotion in different situations (Guo et al., 2017; Henke et al., 2017; Cao et al., 2018b; Duggan et al., 2019; Gamus et al., 2020). The key point is to apply differential friction forces on two sides of the robot body along with its cyclical actuation process. Specifically, for the contracting stage and the extending stage in each actuation cycle, different friction force statuses exist, and then a net locomotion is generated by the asymmetric movements. This can be achieved by controlling either the contacting force (Gu et al., 2018) or the contacting status (friction coefficient) Vikas et al. (2016). However, despite the intuitive locomotion mechanism, complicated actuation structures and control algorithms are required to coordinate the friction forces with the body deformation rhythm.

Dynamic effects exist in most robotic systems. The dynamic effects are usually with high frequency, large interaction force, and more complex status characteristics (Aguilar et al., 2016). Despite these limitations, properly utilizing dynamic effects in a robotic system can help enhance the system performance or expand the system capability, for example, enabling multidirectional locomotion for an underactuated legged robot (Tang et al., 2019), generating high-efficiency rectilinear locomotion using simple oscillation-based actuation (Li et al., 2019), or enabling multimodal locomotion for a soft dielectric elastomer actuator (DEA) robot using different actuation patterns (Duduta et al., 2020). More research studies can be referred to Saunders et al. (2011), Li et al. (2018), and Wu et al. (2019).

In this study, we aim to utilize the body dynamics of a soft inchworm crawler to expand its locomotion capability, that is, realizing bidirectional locomotion *via* a simple system configuration and minimalist actuation input. To the best of our knowledge, there is no other similar study on achieving such multidirectional locomotion *via* only one single actuation. Some vibration-based control scheme can achieve similar locomotion capability but either with complicate actuation schemes (Zimmermann et al., 2011; Lee and Park, 2014) or require external actuation support (Ding and Ziaie, 2009). While several previous research has studied the one-directional locomotion of inchworm robots (Yang et al., 2016; Zhu et al., 2017), we integrated two kinds of differential friction forces into a beam-like robot structure and proposed two locomotion gaits related to the dynamic deformation process of the robot body. As a result, by assigning opposite locomotion directions for these two locomotion gaits, we can achieve a versatile locomotion result by controlling the body deformation process, in which the deformation of the robot body worked as an “implicit” controller to switch the two locomotion gaits automatically.

**FIGURE 1:**
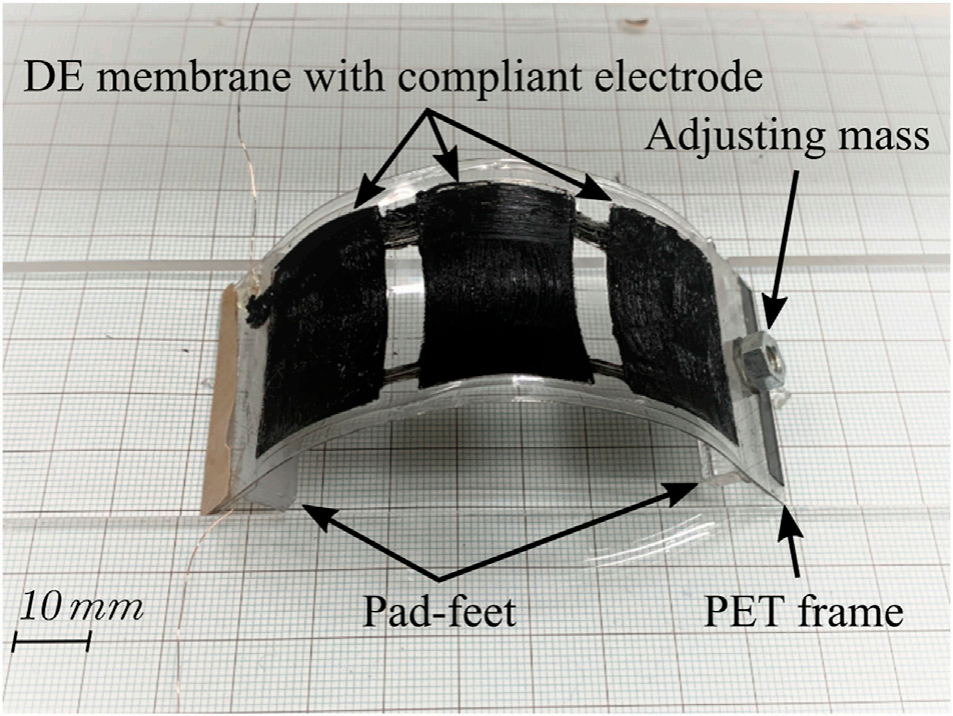
Proposed soft inchworm crawler prototype.

For the rest of this study, first, we present the proposed bidirectional locomotion mechanism in Section 2; then, we propose a simplified mathematical model to explain the robot locomotion process in Section 3; later, we perform a simulation study on the robot model to explore its locomotion performance under different actuation inputs in Section 4; and lastly, we implement the robot mechanism with a DEA-based robot prototype (Figure 1) and perform experimental tests to further validate our proposal in Section 5. Conclusions and future work are discussed in Section 6.

## 2 Principle of Locomotion

### 2.1 Soft Inchworm Crawler Structure

**FIGURE 2:**
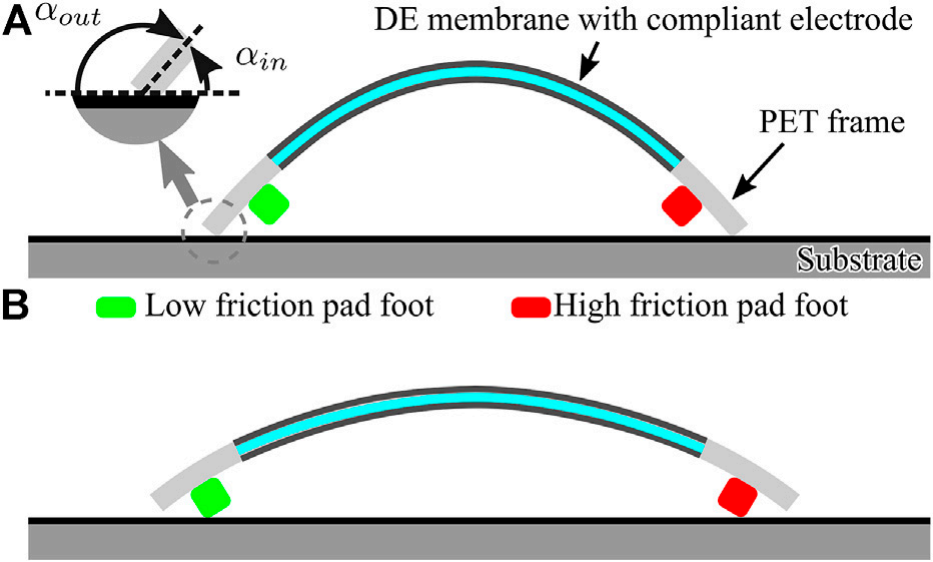
Soft inchworm crawler structure showing two typical body-shape changes and the resulted substrate-contacting statuses. **(A)** Short-length state with body ends contacting the substrate and **(B)** Long-length state with pad-feet contacting the substrate.

As shown in Figure 2, the soft inchworm crawler, that is, the robot has a common-seem curved body shape. The curvature changes of the robot body is usually the actuation result. Various actuation mechanisms can be utilized to generate such body deformation result, such as pneumatic actuation (Majidi et al., 2013), thermal actuation (Yang et al., 2016), electric actuation (Gu et al., 2018), and even mechanical actuation (Vikas et al., 2016). When the robot is placed on a horizontal substrate, two ends of its body will contact with the substrate (due to the gravity effect) and generate a locomotion force when the curved body shape changes. Specifically, two extra pad-feet are attached beneath the curved body as an additional locomotion structure. Ideally, 1) when the robot body is at its short-length state (Figure 2A), it will have large curvature and only two ends of its body will contact the substrate and generate the locomotion force; 2) when the robot body is at its long-length state (Figure 2B), it will have small curvature and only two pad-feet will contact with the substrate and generate the locomotion force. If we use the body length *l* to indicate the robot body deformation result, a critical body length *L*_*c*_ can be observed to distinguish these two substrate-contacting statuses, for which *l* < *L*_*c*_ means short-length state, and *l* > *L*_*c*_ means long-length state.

### 2.2 Bidirectional Locomotion Mechanism

When the robot is cyclically actuated to change its body shape, two locomotion gaits can be observed.

1) *Primitive gait*: When the deformation of the robot body does not exceed its critical body length during the cyclical actuation process, that is, max(*l*) < *L*_*c*_, the robot will only rely on the body ends for locomotion generation. Previous studies have shown that the asymmetric property of the beam-like robot structure can help generate continuous locomotion result for such inchworm robot (Yang et al., 2016); Zhu et al., 2017). Following this idea, we defined a direction-based friction force using the asymmetric geometric property of the robot. Figure 2A shows the magnified contacting status of the body ends with the substrate. The attacking angle (*α*) of the body end is different when the robot moves to different directions, for example, *α*_*out*_ when the body end moves outward and *α*_*in*_ when the body end moves inward. The attacking angle difference will result in different friction coefficients, which is usually larger friction force for larger attacking angle [known as an anchor effect Majidi et al. (2013); Guo et al. (2017)]. Furthermore, when the two body ends of the robot have different masses and/or friction coefficients, the asymmetric geometric property will drive the robot toward the heavier and large friction coefficient side.
2) *Composite gait*: When the deformation of the robot body exceeds its critical body length during the cyclical actuation process, that is, max(*l*) > *L*_*c*_, the robot contacts with the substrate will dynamically switch between the body ends and the pad-feet as a result. Therefore, when selecting materials with different friction coefficients for the pad-feet, an asymmetric friction property can be established as the substrate contacts change. For example, when the pad-foot of one side can generate larger friction force than the body end, it will prevent the returning movement of this side at the retracting stage, thereby resulting a pure locomotion toward the high-friction side. To be noted, the composite gait is generated on the basis of the primitive gait (considering a full actuation cycle), so the locomotion from the asymmetric geometric property and the locomotion from the asymmetric friction status will add together to decide the overall locomotion result of this composite gait.

The “dynamic” of the proposed locomotion mechanism lies in: 1) the dynamic deformation change of the robot under cyclical actuation input and 2) the dynamic integration of different friction mechanisms according to the deformation result of the robot body. When properly designing the robot structure and selecting different friction coefficient materials for its body ends and pad-feet, the proposed inchworm crawler can have two gaits toward different directions at the same time, that is, the bidirectional locomotion result. Moreover, these two dynamic gaits will be automatically activated according to different actuation statuses on the robot body.

Specifically, it should be noted that the key point in this locomotion mechanism is the combination of two kinds of differential friction force using the deformation process of the robot body, so it is independent of the actuation form, that is, the dynamic gaits can be implemented for a variety of robot forms that have similar curved-shaped actuation results.

## 3 Robot Dynamics

In this section, we propose a simplified mathematical model for understanding the soft inchworm crawler that moves on a horizontal plane, which helps explain the locomotion mechanism behind the dynamic process and also predict the locomotion performance of the robot.

### 3.1 Simplification Assumption

To analyze the robot dynamics, we first make several assumptions to simplify the robot structure.

1) The robot is symmetrical along its body-length direction, and the deformation result from the actuation mainly concentrates within its central plane. Therefore, we use a two-dimensional (2D) structure to represent the robot (Figure 2).
2) When the two body ends occupy most of the body mass, the robot can be regarded as two lumped-masses connected together. Then, the robot will perform a 1D movement under the body constraint, actuation input, gravity force, and friction force.
3) The robot central structure is treated as a linear spring, and the actuation input generates proportional actuation force on the two body ends. As a result, the spring length variation will be the actuation result, representing the robot body length changes in the proposed dynamic gaits.

### 3.2 Dynamics Model

**FIGURE 3:**
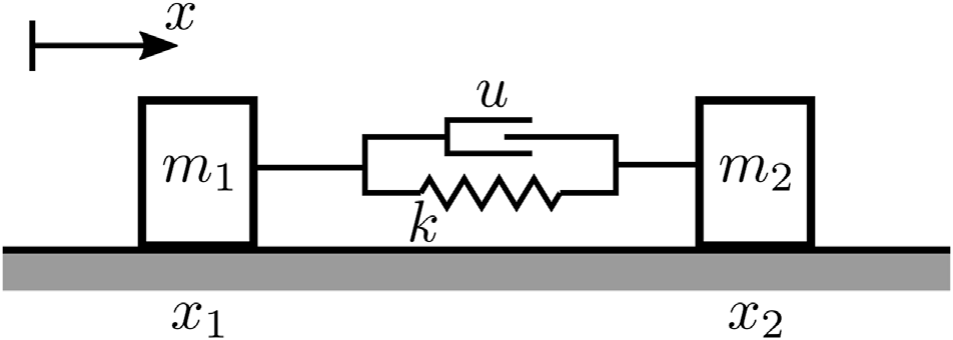
Simplified mathematical model for the soft inchworm crawler.

As shown in Figure 3, the mathematical model for the robot is two masses connected by a passive linear spring and an active actuator, forming a 1D rectilinear locomotion system (Li et al., 2021). Let the spring have a constant stiffness *k* [Nm] and a rest length *L*_0_ [m], the linear actuator generate a force input *u* [N], the two masses have weights *m*_1_ and *m*_2_ [kg], and the differential friction coefficients with the substrate be ${\bar{\mu }}_{1}$ and ${\bar{\mu }}_{2}$ , respectively. The locomotion of the robot can be represented by the position changing of the two masses in a 1D coordinate system, that is, *x*_1_ and *x*_2_ [m]. Therefore, when the masses are measured against their own original position, the motion equation of the system can be defined in a generalized coordinate form as:

$$M\ddot{x}+Kx=Su+F,$$ (1)

in which

$$\begin{array}{ll} & x=\left[\begin{array}{c} x_{1}\\ x_{2} \end{array}\right],M=\left[\begin{array}{cc} m_{1} & 0\\ 0 & m_{2} \end{array}\right],K=\left[\begin{array}{cc} k & -k\\ -k & k \end{array}\right],\\ & S=\left[\begin{array}{c} -1\\ 1 \end{array}\right],F=\left[\begin{array}{c} -m_{1}g{\bar{\mu }}_{1}\mathrm{sign}\left({\dot{x}}_{1}\right)\\ -m_{2}g{\bar{\mu }}_{2}\mathrm{sign}\left({\dot{x}}_{2}\right) \end{array}\right]. \end{array}$$

The key point for realizing the desired bidirectional locomotion lies in controlling the friction coefficients $\left({\bar{\mu }}_{i}\right)$ on each side of the robot body, which is a combination of two kinds of differential friction coefficient using the deformation process of the robot body: 1) the direction-based friction coefficient $\mu_{i}^{\text{forward}}$ and $\mu_{i}^{\text{backward}}$ , which describe a body end friction status $\left(\mu_{i}^{\text{outside}}\right)$ according to its moving direction; 2) the shape-related friction coefficient $\mu_{i}^{\text{outside}}$ (body end) and $\mu_{i}^{\text{inside}}$ (pad-foot), which describe a compound friction status on each side of the robot according to its contacting status with the substrate. First, we can model the body end friction coefficient as:

$$\mu_{i}^{\text{outside}}=\frac{\mathrm{sign}\left({\dot{x}}_{i}\right)+1}{2}\mu_{i}^{\text{forward}}+\frac{-\mathrm{sign}\left({\dot{x}}_{i}\right)+1}{2}\mu_{i}^{\text{backward}},\;i=1,2,$$ (2)

in which $\mu_{i}^{\text{outside}}$ equals either $\mu_{i}^{\text{forward}}$ or $\mu_{i}^{\text{backward}}$ according to the body-end sliding direction. Second, by integrating the body-end friction coefficient $\mu_{i}^{\text{outside}}$ with the pad-foot friction coefficient $\mu_{i}^{\text{inside}}$ using a shape-related combining function, we can get a compound friction coefficient result for each side of the robot as:

$${\bar{\mu }}_{i}=\frac{\mathrm{sign}\left(x_{2}-x_{1}+L_{0}-L_{c}\right)+1}{2}\mu_{i}^{\text{inside}}+\frac{-\mathrm{sign}\left(x_{2}-x_{1}+L_{0}-L_{c}\right)+1}{2}\mu_{i}^{\text{outside}},i=1,2,$$ (3)

in which *l* = *x*_2_ − *x*_1_ + *L*_0_ will be the actual robot body length and ${\bar{\mu }}_{i}$ will either equal to the body-end friction coefficient $\mu_{i}^{\text{outside}}$ when *l* < *L*_*c*_ or equal to the pad-foot friction coefficient $\mu_{i}^{\text{inside}}$ when *l* > *L*_*c*_.

## 4 Simulation Study

Due to the involvement of the two differential friction forces, the proposed mathematical model for the robot is highly non-linear and difficult to be solved analytically. In this section, we use numerical simulations to explore the locomotion behavior of the robot.

### 4.1 Locomotion Generation

The parameters used for the numerical simulations are listed in Table 1, which are chosen based on the robot prototype developed in Section 5. Specifically, we simplify the direction-based friction force on body ends with an assumption that the movement toward small attacking angle direction will experience half of the full friction force, that is, $\mu_{1}^{\text{forward}}$ is half of $\mu_{1}^{\text{backward}}$ for the backward body end, and $\mu_{2}^{\text{backward}}$ is half of $\mu_{2}^{\text{forward}}$ for the forward body end.

**TABLE 1:** Robot parameters in the simulation study.

—	—	—
Backward body mass	*m*_1_	0.003 kg
Forward body mass	*m*_2_	0.002 kg
Backward body-end friction coefficient	$\mu_{1}^{\text{forward}}$ , $\mu_{1}^{\text{backward}}$	0.05, 0.1
Forward body-end friction coefficient	$\mu_{2}^{\text{backward}}$ , $\mu_{2}^{\text{forward}}$	0.1, 0.2
Backward pad-foot friction coefficient	$\mu_{1}^{\text{inside}}$	0.1
Forward pad-foot friction coefficient	$\mu_{2}^{\text{inside}}$	0.3
Spring stiffness	*k*	0.7 Nm
Rest state body length	*L*_0_	0.05 m
Fully actuated state body length	*L*_1_	0.08 m
Critical body length	*L*_*c*_	0.07 m

In the simulation study, the actuation input *u* is set in a sine waveform to simulate a cyclical actuation process:

$$u=A\cdot k\left(L_{1}-L_{0}\right)\cdot \frac{\sin\left(2\pi ft\right)+1}{2},$$ (4)

in which *A*(0 ≤ *A* ≤ 1) is the nominated actuation force amplitude^1^ that ensures the chosen rest state body length (*L*_0_) and the fully actuated body length (*L*_1_) and *f* is the actuation force frequency. For each simulation, a steady-state locomotion velocity $\bar{v}$ is calculated as:

$$\bar{v}=\frac{x_{i}\left(t+1/f\right)-x_{i}\left(t\right)}{1/f},\;i=1,2.$$ (5)

**FIGURE 4:**
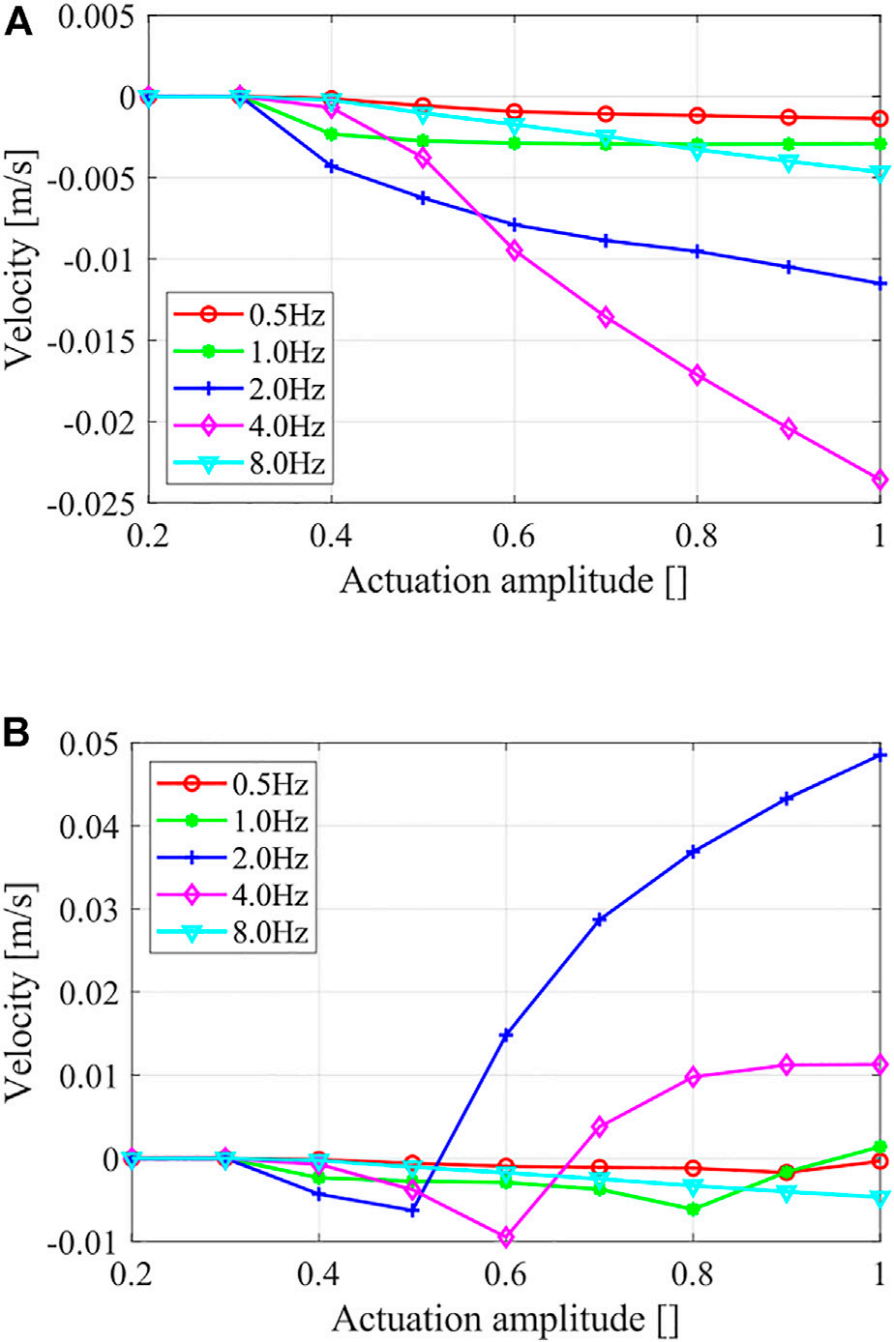
Steady-state locomotion velocities show the simulation results under different actuation magnitudes and frequencies. **(A)** Locomotion with only direction-based friction force and **(B)** Locomotion with a compound friction force.

We first verified the robot locomotion with only the direction-based friction force, that is, the “primitive gait,” in Section 2. Figure 4A shows the steady-state velocity of the locomotions generated under different actuation magnitudes and frequencies. Note that: 1) the robot achieved unified locomotion direction under different actuation magnitude and frequency combinations; 2) the steady-state velocity increased along with the actuation magnitude; 3) the steady-state velocity dropped for higher actuation frequency; and 4) resonance effect occurred around an actuation frequency of 4 Hz, resulting abnormally high locomotion velocity.

**FIGURE 5:**
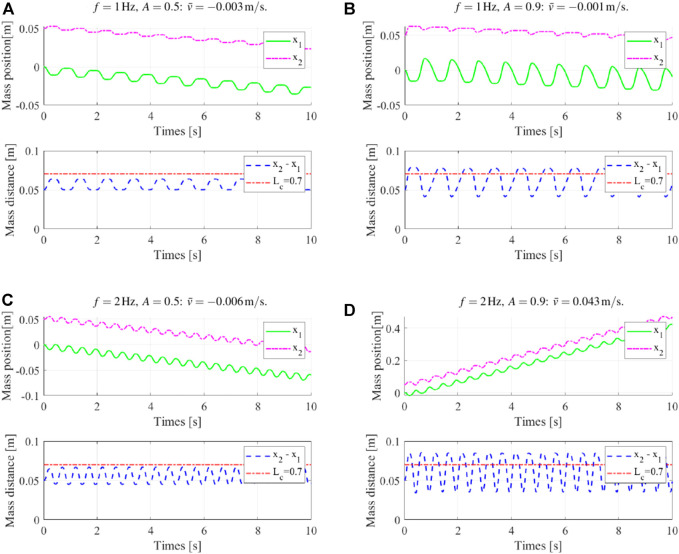
Typical locomotion results using the compound friction force. **(A)** and **(B)** “composite gait” weakening the “primitive gait” locomotion result, while it has not reversed the locomotion direction; **(C)** and **(D)** “composite gait” reversing the “primitive gait” locomotion result.

We then studied the robot locomotion with the compound friction force, that is, the bidirectional locomotion using the proposed dynamic gaits. A comparison of the locomotion results under different actuation magnitudes and frequencies using the compound friction force is shown in Figure 4B, and the details of four typical locomotion results are shown in Figure 5. Generally, the previous unified locomotion direction got changed due to the introduction of the compound friction force, that is, for certain actuation frequency ranges (e.g., 2 Hz and 4 Hz), the robot kept its locomotion direction when at small actuation magnitude but reversed its locomotion direction when at larger actuation magnitude. Furthermore, the details of locomotion results in Figure 5 validated the effectiveness of the “composite gait” in altering the locomotion result, which happened when the robot body length variations surpass its critical body length *L*_*c*_. Lastly, these results also validated the dynamic gait control scheme, that is, a frequency-based locomotion control comparing Figures 5B,D and magnitude-based locomotion control comparing Figure 5C and Figure 5D. Therefore, instead of directly controlling the friction forces, the proposed “implicit controller” controls the deformation amplitude and frequency of the robot *via* choosing proper actuation input, as shown in Eq. 4; then, the combination of the two differential friction forces and the locomotion of the robot is indirectly controlled.

### 4.2 Locomotion Performance

**FIGURE 6:**
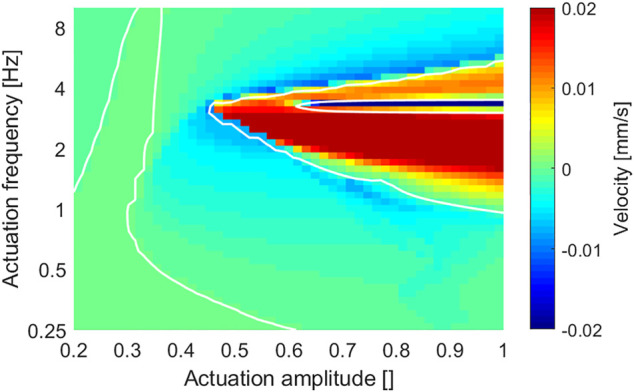
Overview of the robot locomotion velocity under different actuation amplitudes and frequencies. 1) Overall velocity range is suppressed to show more low-velocity details; and 2) white contour line shows the zero-velocity separation.

Figure 6 presents an overview of the robot locomotion velocity under different actuation amplitudes and frequencies. The white contour line of zero velocity helps divide the whole map into positive velocity regions and negative velocity regions. Note that: 1) the locomotion velocity was small and negligible when at lower actuation amplitude (*A* < 0.35) or at lower actuation frequency (*f* < 0.5 Hz); 2) the basic locomotion results were toward the negative (backward) direction, and the positive (forward) direction results were mainly resulted from large actuation magnitude with moderate actuation frequency; and 3) the abnormal large velocity zone existed around 3 Hz, resulted from a resonance effect. This velocity map can be used to predict results of different dynamic-gait control strategies, for example, a vertical trajectory for frequency-based control and a horizontal trajectory for amplitude-based control.

Cost of transport (CoT) is one commonly used method to evaluate the energy efficiency of mobile robots. It is the actuation energy required for the robot to travel a certain distance (usually 1 kg mass and 1 m distance) (Full and Tu, 1991; Srinivasan and Ruina, 2006). Generally, a small CoT value means higher locomotion efficiency. For the proposed robot, the CoT in one actuation cycle at a steady state is calculated as follows:

$$CoT=\frac{\int_{t}^{t+1/f}max\left\{{\dot{x}}^{\text{T}}Su,0\right\}dv}{\left(m_{1}+m_{2}\right)g|x_{i}\left(t+1/f\right)-x_{i}\left(t\right)|},\;i=1,2.$$ (6)

**FIGURE 7:**
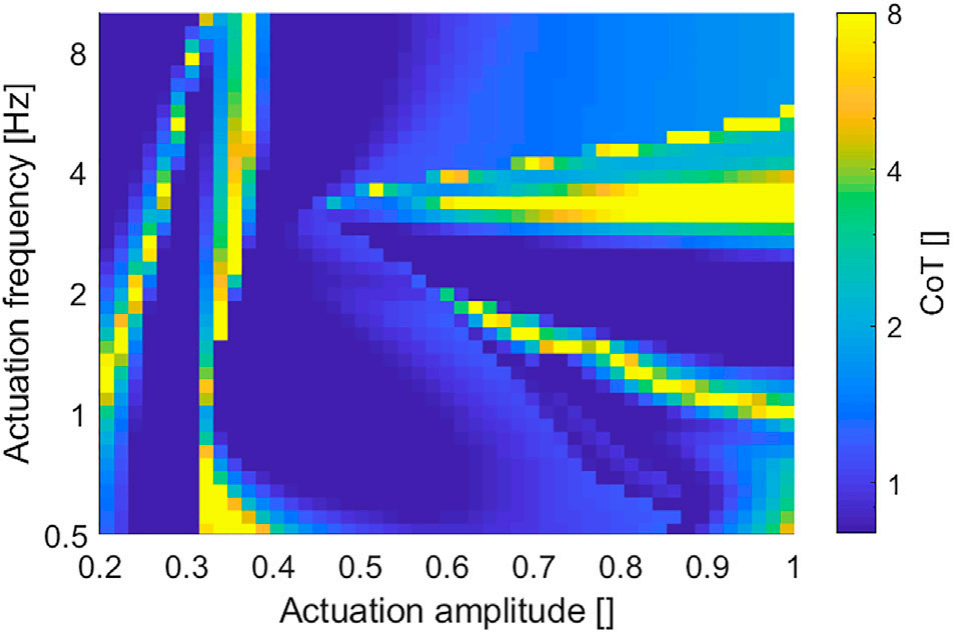
Overview of the robot locomotion CoT under different actuation amplitudes and frequencies (the overall CoT range is suppressed to show more low CoT details).

Figure 7 presents an overview of the CoT under different actuation amplitudes and frequencies. The robot achieved relatively high energy efficiency (*CoT* < 1) in a large part of the result, while the low actuation magnitude region had relatively low energy efficiency. In addition, by comparing with the corresponding velocity result map (Figure 6), the CoT got a notable high value around the contour line of zero velocity, which is understandable considering its definition in Eq. 6.

## 5 Prototype Experiments

In this section, we implemented the robot mechanism into a DEA-based robot prototype and performed locomotion tests to further validate the dynamic-gait control method.

### 5.1 Prototype Fabrication

The robot prototype is based on a DEA structure, which is a kind of novel soft actuation technology that shows large deformation, high power density, and high energy efficiency when compared with the traditional actuation methods Gu et al. (2017); Xu et al. (2017); Gupta et al. (2019); Tiefeng Li et al. (2019). The DEA structure will apply to the soft actuation behavior presented in Section 2 when neglecting non-linear effects Cao et al. (2018a). Still, it should be noted that although the inchworm crawler mechanism can be implemented into a lot of soft robot forms or even rigid robot forms that generate similar structure deformation results, the DEA structure is one desirable choice considering its specialties of lightweight, large deformation, and high dynamic performance, possibly achieving better locomotion performance with a cyclical deformation result Petralia and Wood (2010); Duduta et al. (2017); Guo et al. (2020).

**FIGURE 8:**
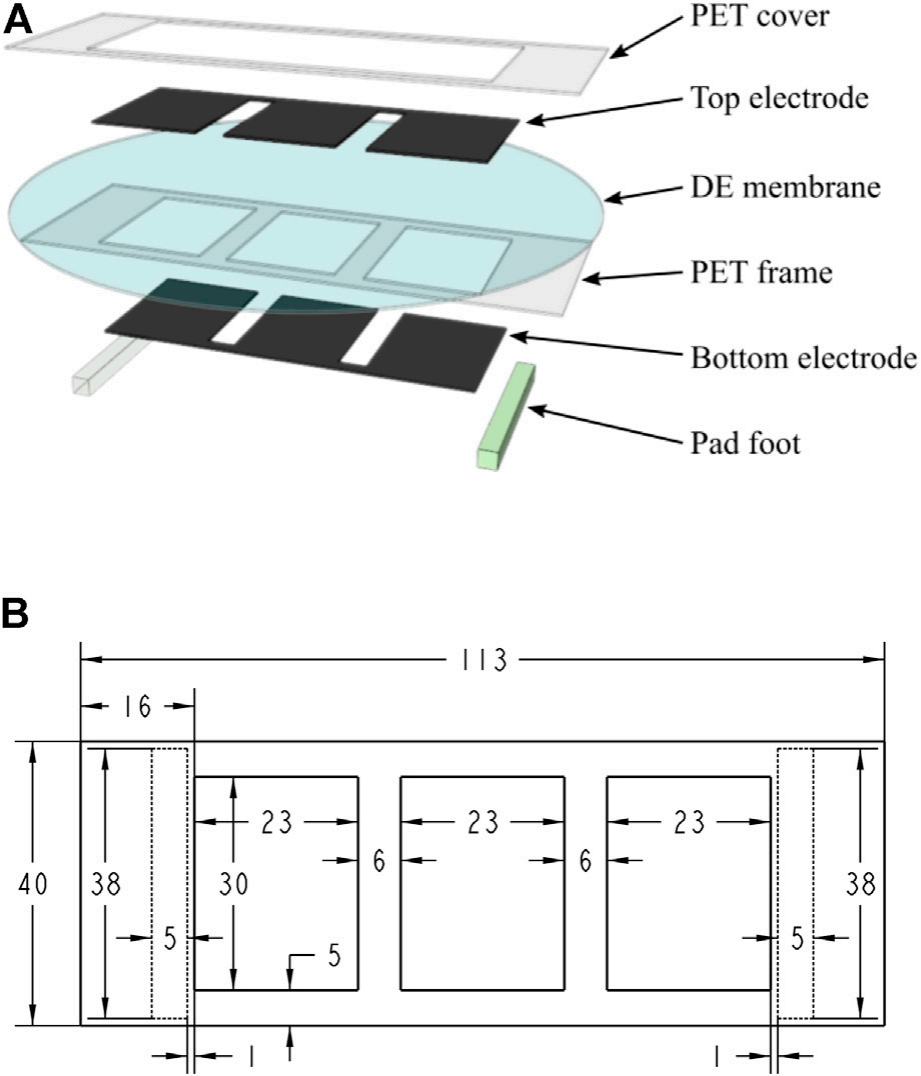
Design of the robot prototype. **(A)** Exploded view of the robot showing the multilayered DEA structure; **(B)** design parameters used for the PET frame and pad-feet (pad-feet shown as dashed line) (dimensions in [mm]).

The developed robot prototype adopted a common-seen planar DEA structure (Gu et al., 2018; Zhang et al., 2018), which is easy-fabrication, and can generate the desired cyclical deformation result for inchworm locomotion generation. Figure 8A shows the multilayered structure of the robot prototype: 1) the DE membrane used one acrylic elastomer (VBH4910, 3M, US) with a pre-stretch ratio of 4 (original thickness 1 mm); 2) the compliant electrode used one carbon conductive grease (846-80G, MG Chemicals, Canada); 3) the PET frame and PET cover were laser-cut from one high elasticity PET board (thickness 0.3 mm). Specially for the bidirectional soft inchworm crawler: 1) we carefully designed the PET frame to get this three-DEA-unit structure (Figure 8B), which can provide a large and stable deformation result within the restricted DE membrane area; 2) the two pad-feet were one acrylic plastic material and one silicon rubber material, providing large friction coefficient difference for locomotion generation; 3) two kinds of plastic tape were wrapped on each side of the PET frame to create friction coefficient difference on the body ends, for which the acrylic pad-foot side has relatively small friction coefficient; and 4) the additional mass of 1.5 g was added on the acrylic foot side. After finishing the multilayered structure, it was cut off from the circular DE membrane to form the curve-shaped robot prototype (Figure 1). The PET frame and DE membrane weighed about 1.39 g, the rubber foot weighed about 1.35 g, and the acrylic foot weighed about 1.04 g. The two body ends account for about 85% of the whole robot mass, while the DEA structure takes 15% of the whole robot mass, which is consistent with the model simplification assumptions in Section 3.1. Table 2 shows other key parameters of the fabricated robot prototype.

**TABLE 2:** Key parameters of the robot prototype.

—	—
Left body-end friction coefficient	0.05
Right body-end friction coefficient	0.1
Left pad-foot friction coefficient	0.4
Right pad-foot friction coefficient	0.1
Rest state body length	0.085 m
Fully actuated state body length	0.105 m
Critical body length	0.095 m

### 5.2 Experiment Setup

To actuate the robot prototype, we developed a customized actuation circuit that can generate programmable high-voltage sequences for DEA actuation. The DEAs were actuated with on–off states. First, we used a high-voltage DC (HVDC) converter (FS50P-12, XP Power, US) to generate a voltage adjustable high-voltage source (up to 5 kV). Then, we used a microcontroller board (Arduino Mega 2560) to generate the on–off signal with controllable actuation frequency, which was amplified by intermediate signal relays (V23026, TE Connectivity, US) and then led to high-voltage relays (DAT71215F-HR, Cynergy3 Components, UK) to control the charging and discharging process of the DEAs in the robot. As a result, the circuit can provide up to *U*_full_ = 5 kV on–off actuation voltage sequence with an actuation frequency up to 5 Hz (limited by the relay’s response time).

**FIGURE 9:**
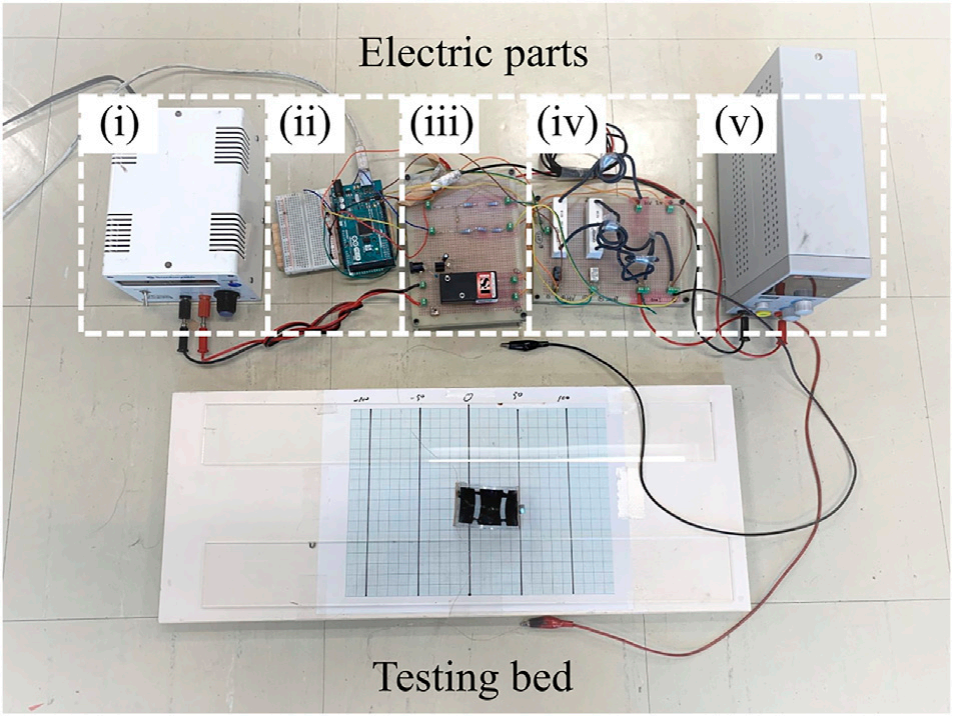
Overview of the whole experiment setup showing the electric parts and the robot locomotion testing bed. (i) Adjustable input voltage for the HVDC converter; (ii) Arduino Mega; (iii) HVDC converter; (iv) relay board; and (v) relay power supply.

We placed the robot prototype on a horizontal testing bed and connected it to the actuation circuit *via* *ϕ*0.2-mm copper wires. In addition, a coordinate paper was placed under to robot to help read the locomotion result, and two acrylic boards were placed aside of the robot as guide rails. An overview of the whole experiment setup is presented in Figure 9. The high voltage applied on DEA was estimated based on the HVDC converter datasheet.

### 5.3 Experiment Result

**FIGURE 10:**
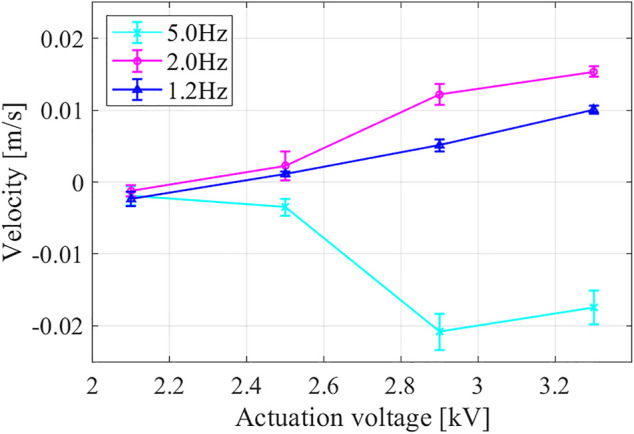
Prototype steady-state velocities under different actuation magnitudes and frequencies.

We first tested the robot locomotion under different actuation magnitudes and frequencies: the minimum voltage (around 2.1 kV) is chosen to ensure a noticeable cyclical actuation result, and the maximum voltage has to ensure the DEA structure safety; the minimum frequency (around 1 Hz) is chosen to ensure continuous deformation process, and the maximum frequency is restricted by the relay response times. The robot generated continuous locomotion under different actuation statuses, and the locomotion direction can be controlled by changing the actuation to certain magnitudes and frequencies. Figure 10 shows measured steady-state velocities under different actuation magnitudes and frequencies, in which each measurement was performed five times to get a statistical result. It can be noticed that: 1) for smaller actuation magnitude, locomotion at different actuation frequencies all achieved backward results due to the primitive gait; 2) when the actuation magnitude was increased, locomotion at lower actuation frequencies (1.2 and 2.0 Hz) reversed their locomotion direction due to the involvement of the shape-related friction effects, while locomotion at higher frequency (5 Hz) kept its locomotion direction. However, a discrepancy between the simulation result and prototype experiment does exist, which can be attributed to: 1) the simplification of the robot model, 2) the conceptualization of the friction status, and 3) the inaccuracies in system parameters.

**FIGURE 11:**
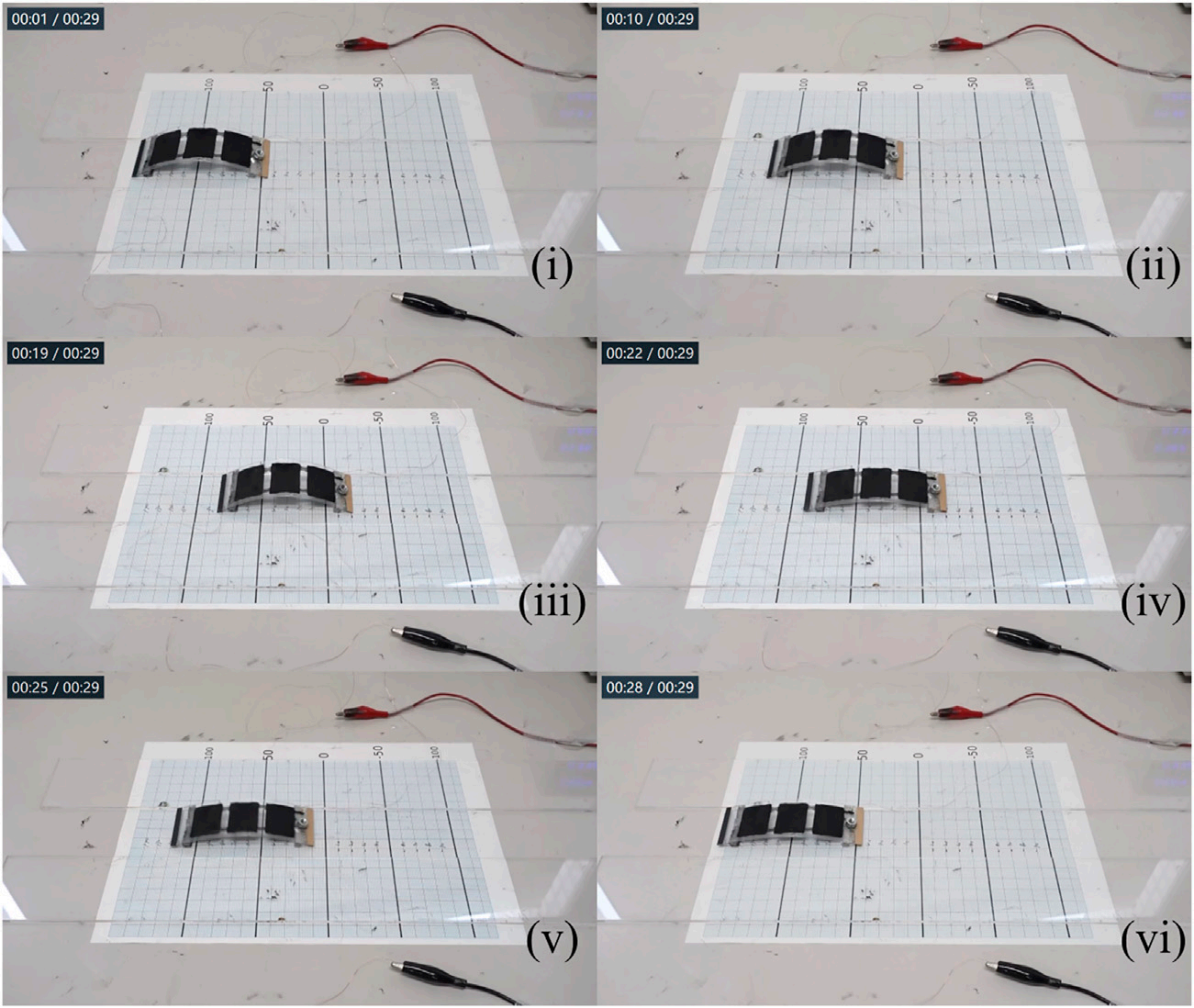
Snapshot images from the voltage-based locomotion control demo: the actuation frequency was 2 Hz; the actuation voltage in (i), (ii), and (iii) was 2.1 kV, and in (iv), (v), and (vi) was 3.3 kV.

Based on the robot locomotion velocity results, we validated the dynamic-gait control method with two locomotion demos, one voltage-based locomotion control, and one frequency-based locomotion control, in which the robot first achieved a continuous locomotion result toward one direction and then reversed its locomotion direction when the actuation voltage magnitude or frequency was adjusted. A complete video showing the dynamic-gait control method is provided as Supplementary Material in the Appendix section. In addition, Figure 11 shows snapshot images from the voltage-based locomotion control demo. The actuation frequency was 2 Hz. The robot moved toward the backward direction when the actuation voltage was about 2.1 kV and then reversed to the forward direction when the actuation voltage was increased to about 3.3 kV. It should be noted that the robot body extended to a longer body-length at higher actuation voltage [(iv), (v), and (vi) in Figure 11], which helped the robot switch to the composite gait from the initial primitive gait.

### 5.4 Discussion

Although the proposed inchworm crawler mechanism can be implemented into a lot of soft robot forms or even rigid robot forms, proper structural parameter combinations and control inputs are necessary to get a desirable bidirectional locomotion result. We can also infer that the environment will strongly affect the locomotion performance, for example, a different substrate surface condition may result in different friction status and change the robot locomotion performance; for a tilted surface, the gravity component along the moving direction may break the differential-friction-force relationships and affect the bidirectional locomotion result; for a curved surface, further study will be necessary to explore the changes to the proposed robot mechanism and the effects resulted.

## 6 Conclusion and Future Work

This study has presented a soft inchworm crawler that achieved bidirectional locomotion capability by using a dynamic-gait control method. We implemented two differential friction mechanisms into a beam-like soft robot structure. Two locomotion gaits can be generated at different cyclical deformation magnitudes of the robot body, and we can control the robot locomotion result by using its body deformation process as a built-in “implicit controller.” A simplified mathematical model was proposed to analyze the robot locomotion process, and numerical simulation studies were carried out to validate the proposed dynamic-gait control method, which successfully showed the possibility of both amplitude-based control and frequency-based control of the robot. Also, a robot prototype using a DEA structure was fabricated, and experiments were performed that further validated the dynamic-gait control method for bidirectional locomotion. The soft inchworm crawler achieves versatile locomotion results with a simple system configuration and minimalist actuation input. The locomotion mechanism can be implemented for a variety of robot forms to meet different application requirements. Moreover, the effective utilization of dynamic effects in the soft robot structure is foreseen to be inspiring for further performance promotion of the existing soft robotic research studies.

Future work will perform robot dynamics study to better understand the inchworm locomotion mechanism. Also later, improved robot design and use of advanced control schemes will be considered.

## Data Availability

The datasets generated for this study are available on request to the corresponding author.

## Footnotes

1 The nominated actuation input simplifies the analysis content and generalizes the conclusion regardless of specific robot parameters.
